# Astronomical age constraints and extinction mechanisms of the Late Triassic Carnian crisis

**DOI:** 10.1038/s41598-017-02817-7

**Published:** 2017-05-31

**Authors:** Charlotte S. Miller, Francien Peterse, Anne-Christine da Silva, Viktória Baranyi, Gert J. Reichart, Wolfram M. Kürschner

**Affiliations:** 1 Department of Geosciences, University of Oslo, Oslo, 0371 Norway; 2 Department of Earth Sciences, Utrecht University, Utrecht, Netherlands; 3 Department of Geology, University of Liège, Liège, Belgium; 4 Department of Ocean Systems, Royal Netherlands Institute for Sea Research, ‘t Horntje, Netherlands; 5 MARUM Center for Marine Environmental Sciences, University of Bremen, Bremen, Germany

## Abstract

The geological record contains evidence for numerous pronounced perturbations in the global carbon cycle, some of which are associated with mass extinction. In the Carnian (Late Triassic), evidence from sedimentology and fossil pollen points to a significant change in climate, resulting in biotic turnover, during a time termed the ‘Carnian Pluvial Episode’ (CPE). Evidence from the marine realm suggests a causal relationship between the CPE, a global ‘wet’ period, and the injection of light carbon into the atmosphere. Here we provide the first evidence from a terrestrial stratigraphic succession of at least five significant negative C-isotope excursions (CIE)’s through the CPE recorded in both bulk organic carbon and compound specific plant leaf waxes. Furthermore, construction of a floating astronomical timescale for 1.09 Ma of the Late Triassic, based on the recognition of 405 ka eccentricity cycles in elemental abundance and gamma ray (GR) data, allows for the estimation of a duration for the isotope excursion(s). Source mixing calculations reveal that the observed substantial shift(s) in δ^13^C was most likely caused by a combination of volcanic emissions, subsequent warming and the dissociation of methane clathrates.

## Introduction

The Late Triassic period represents a time of extreme aridity and relative environmental and climatic stability, interrupted only by a brief, but substantial switch to more humid conditions, followed by ecological crises in the middle Carnian, during the CPE^1–5^. The CPE is characterised by elevated extinction rates in the marine realm, and a contemporaneous increase in terrestrial species diversity with a temporary switch to more hygrophytic flora^6–8^. Subsequently, the late Carnian marks the dawn of calcareous nanoplankton and scleractinian reef builders and the rise of the early dinosaurs^9^.

**Figure 1:**
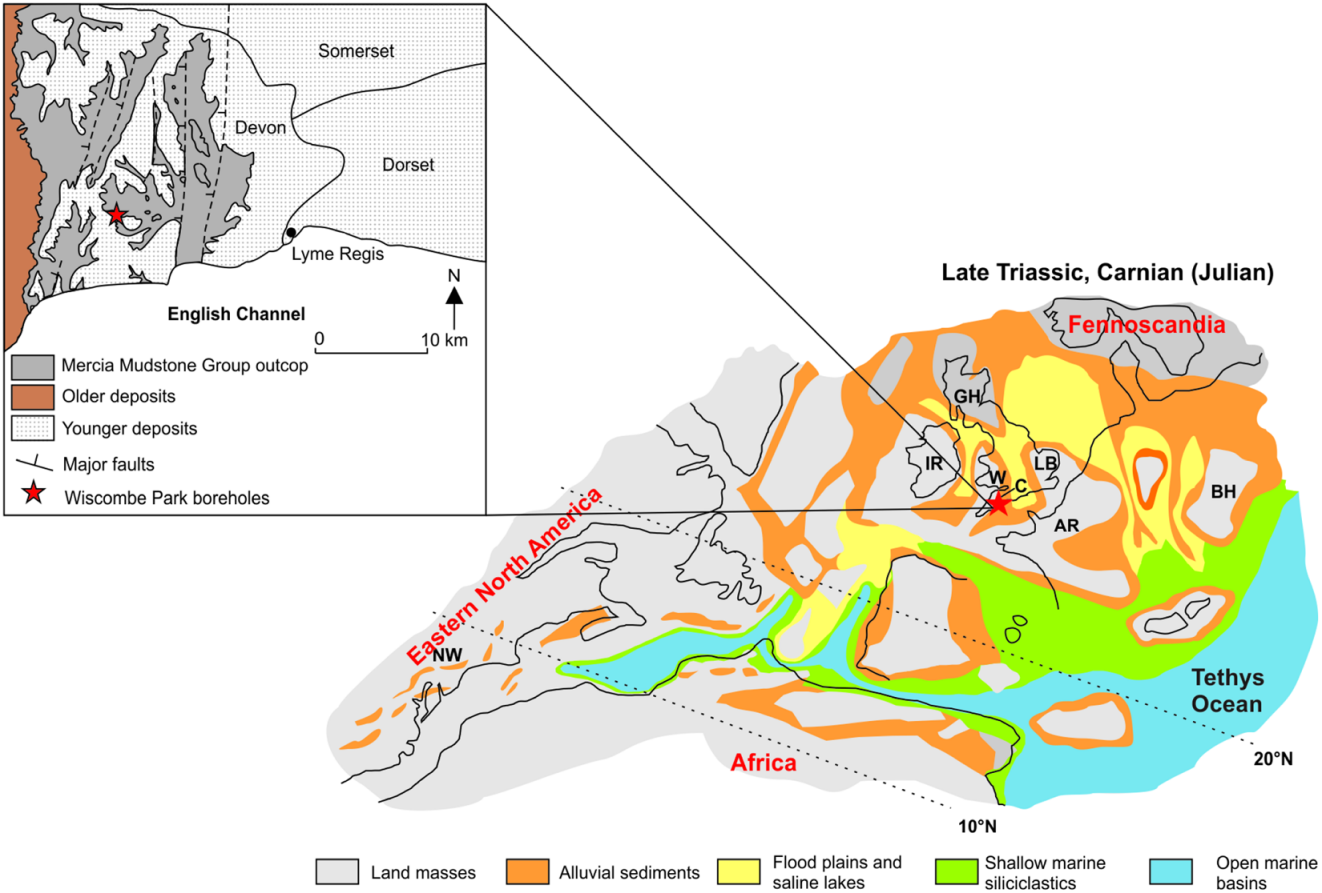
Palaeogeographic map of western Europe, eastern North America and Africa during the Carnian adapted from^44^, using CorelDraw Graphics Suite X7 (http://www.coreldraw.com/de/) Copyright (c) 2015 [MARUM] and its licensors. All rights reserved. Insert shows the distribution of the Mercia Mudstone Group and the location of the WP boreholes. Abbreviations: GH (Grampian High), IR (Ireland), W (Wales), C (Cornubia), AR (Armorica), LB (London Basin), BH (Bohemian-Vindicelian High) and NW (Newark Basin).

In terrestrial successions of the Carnian in southwest England (Fig. 1), red evaporitic mudstones and siltstones of the Sidmouth Mudstone Formation (SMF), representing hyper-saline sabkha sedimentation, are replaced by lacustrine green-grey mudstones and dolomitic limestones of the Dunscombe Mudstone Formation (DMF) suggesting a widespread increase in precipitation during the CPE (middle Carnian)^10–15^. Contemporaneously, in the Germanic Basin, sabkha sediments are replaced by predominantly fluvial deposits of the Stuttgart Formation (Schilfsandstein)^16^. Furthermore, marine records indicate the establishment of oxygen depleted conditions in marginal basins, with an increase in siliciclastics, and a temporary shutdown of carbonate systems across the Tethyan realm (Fig. 1)^17^.

Despite the global significance of the CPE as a major environmental perturbation, the trigger of the environmental change and subsequent biotic turnover is still disputed. Evidence from the marine realm of the existence of both a bulk (*c*. 2‰) and a compound specific (4‰) negative CIE at the onset of the CPE suggests the injection of ^13^C-depleted CO_2_ into the Earth system^3^. Nevertheless, no carbon isotopic evidence for the CPE from a terrestrial stratigraphic succession exists. Moreover, the timing and duration of the CPE is still unclear thereby hampering the establishment of a causal relationship between the mechanism, the CIE, and the associated environmental change. Here we present for the first time continental records of: i) bulk sediment δ^13^C, and ii) compound specific δ^13^C of plant leaf waxes (weighted mean of C_27_-C_35_ *n*-alkanes; δ^13^C_wax_) through the Late Triassic (Carnian) of the Wiscombe Park borehole (WP; Devon, UK), to assess the CPE within the terrestrial realm. Additionally, cyclostratigraphic investigation using elemental abundance and GR data is used to establish a floating time-scale for the succession, thus constraining the duration of the CIEs.

Samples were collected from WP borehole 1 [SY 1819 9382], and GR data from WP borehole 2^10^ [SY 1845 9273] (Fig. 1), currently stored at the British Geological Survey (UK). Palynomorph assemblages indicate that the DMF is Carnian (Julian 2) in age (see supplementary information). Therefore, we interpret the lithological change at the base of the DMF (marking the switch to a lacustrine environment) to be concomitant with the environmental turnover found in marine realm during the CPE at the Julian 1–2 boundary^2^.

**Figure 2:**
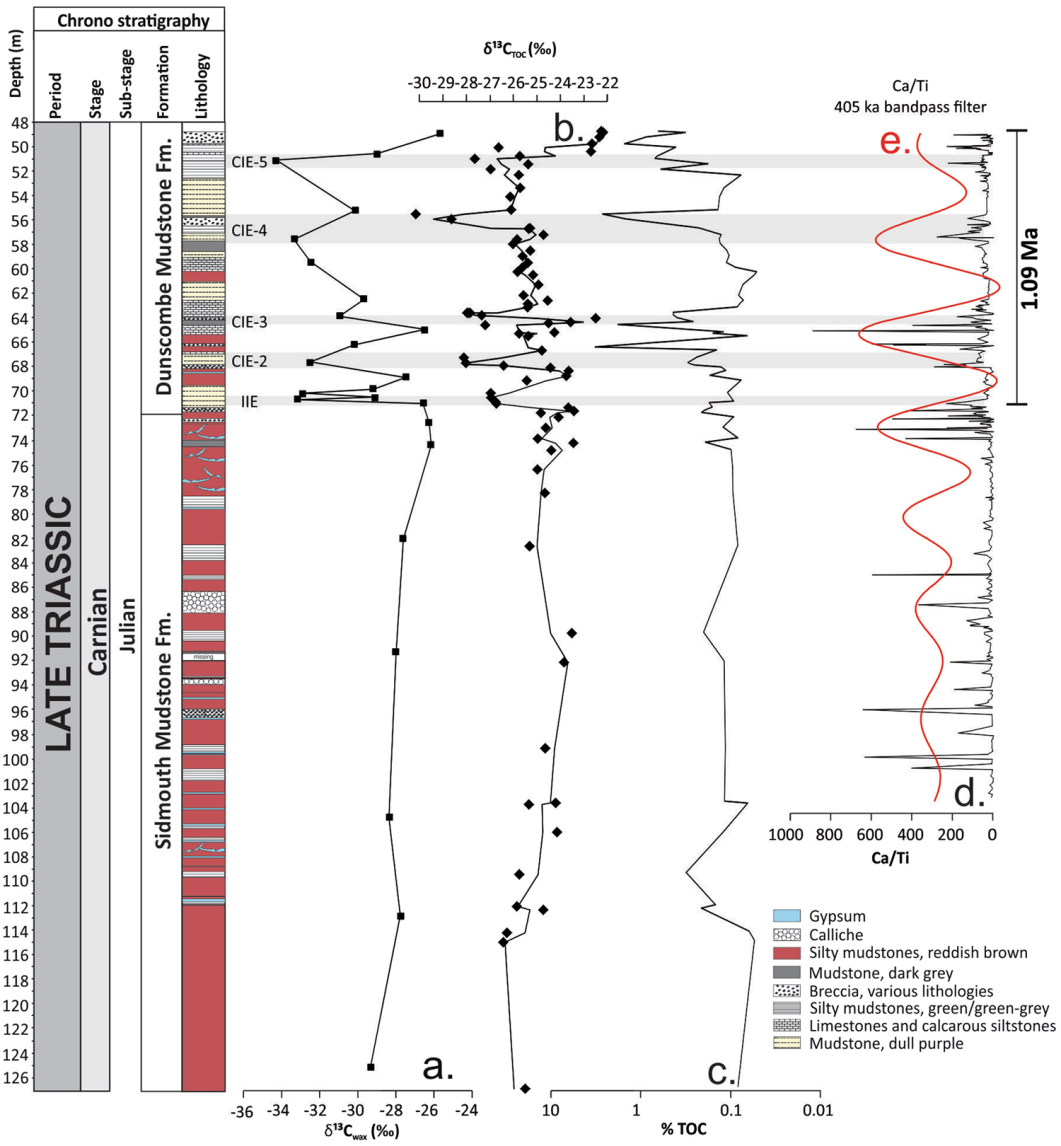
Environmental variability over the CPE from WP borehole 1. (**a**) Weighted mean of δ^13^C values for long chain *n*-alkanes (C_27_-C_35_; δ^13^C_wax_). (**b**) δ^13^C variations in bulk organic matter (δ^13^C_TOC_). (**c**) Percentage total organic carbon (TOC). (**d**) XRF Ca/Ti elemental abundance data. (**e**) 405 ka bandpass filter of the Ca/Ti XRF data. Note the dominant cyclicity present at *c*. 8 m, likely corresponding to the 405 ka eccentricity cycle (see also Fig. 3 and Fig. S6). The strength of the 405 ka eccentricity control on sedimentation decreases down core. The centre of the bandpass frequency is 0.13 m^−1^ (*c*. 8 m, *c*. 405 ka). Data filtering was carried out using a Gaussian filter in the R program Astrochron^43^.

The total organic carbon (TOC) contents varies between 0.04 and 7.7% (Fig. 2c), and the bulk carbon isotopic composition (δ^13^C_TOC_) ranges from −30.1‰ to −25.1‰ (Fig. 2b). The δ^13^C_TOC_ indicates relative C isotopic stability through the SMF (*c*. −25‰) but during the DMF fluctuates highly (Fig. 2b). δ^13^C_TOC_ reveals an initial sudden and pronounced negative isotope shift of *c*. 3–4‰ at 71–70.5 m, at the beginning of the DMF, coinciding with a lithological shift indicating wetter conditions. The initial isotope excursion (IIE) is followed by a further four transitory negative CIEs within the DMF, with a return to heavier values of *c*. −22.3‰ from 50 m to the core top (Fig. 2b). Since the composition of TOC (and thus its carbon isotopic composition) can change over time, and is sensitive to (microbial) degradation, we use the carbon isotopic composition of plant leaf waxes (δ^13^C_wax_; see supplementary information) to confirm the trends of the δ^13^C_TOC_ record. Leaf waxes, showing a clear odd-over-even predominance, are produced by higher plants and considered stable once buried in sediments^18^. Hence, variations in their isotopic composition unambiguously record past climate variability^19^ (see supplementary information). δ^13^C_wax_ values range from −25.7‰ to −34.3‰ and are depleted by *c*. 5‰ with respect to bulk δ^13^C_TOC_ (Fig. 2a). The IIE of *c*. 3–4‰ revealed in the δ^13^C_TOC_ is also observed in the δ^13^C_wax_ values at 71–70.5 m; however, the shift is larger *c*. 6–7‰ (Fig. 2). The δ^13^C_wax_ record provides evidence for a further four transitory negative CIEs within the DMF, returning to heavier values of *c*. −26‰ from 49 m to the top of the core (Fig. 2a). Although on initial inspection the δ^13^C_TOC_ data over the CPE appears relatively noisy, a comparison between the equivalent samples measured for δ^13^C_TOC_ and δ^13^C_wax_ show good covariance (supplementary Fig. S4). Similar to the records from the marine successions^3, 20^, we observe a slight long-term increase in δ^13^C trends (*c*. 2‰, in both δ^13^C_wax_ and δ^13^C_TOC_) through the SMF during the Carnian. This trend has been linked to the re-emergence of coal swamps and increased carbon burial rates^20^.

**Figure 3:**
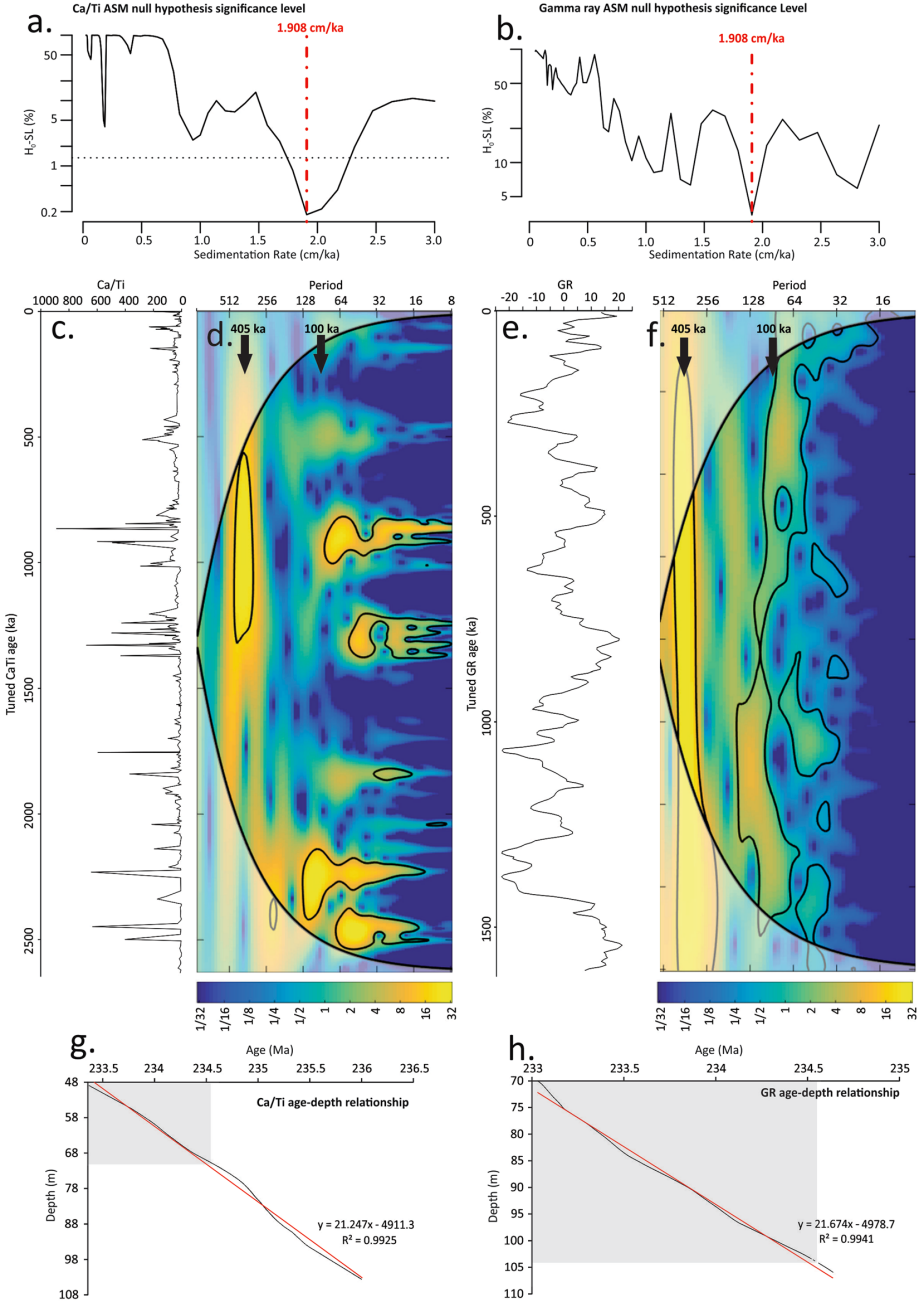
Time series analysis of the Ca/Ti XRF data (all data) and GR data spanning the DMF. (**a**) Sedimentation rate null hypothesis significance levels for Ca/Ti data through the DMF, note the most likely sedimentation rate is 1.908 cm ka^-1^. (**b**) Sedimentation rate null hypothesis significance levels for GR data through the DMF, note the most likely sedimentation rate is 1.908 cm ka^-1^. (**c**) Tuned Ca/Ti data on the 405 ka cycle. (**d**) Wavelet analysis applied on the tuned Ca/Ti dataset. (**e**) Tuned GR data on the 405 ka cycle. (**f**) Wavelet analysis applied on the tuned GR dataset. Pale areas delineate the cone of influence where the wavelet power is uncertain. (**g**) Age-depth relationship for WP borehole 1 (Ca/Ti) derived from tuning to the 405 ka cycle. (**h**) Age-depth relationship for WP borehole 2 (GR) derived from tuning to the 405 ka cycle. Note grey box highlights the DMF in both age-depth plots. The equation in g. is used to construct the floating chronology for the δ^13^C_wax_ and δ^13^C_TOC_ in Fig. 4.

**Figure 4:**
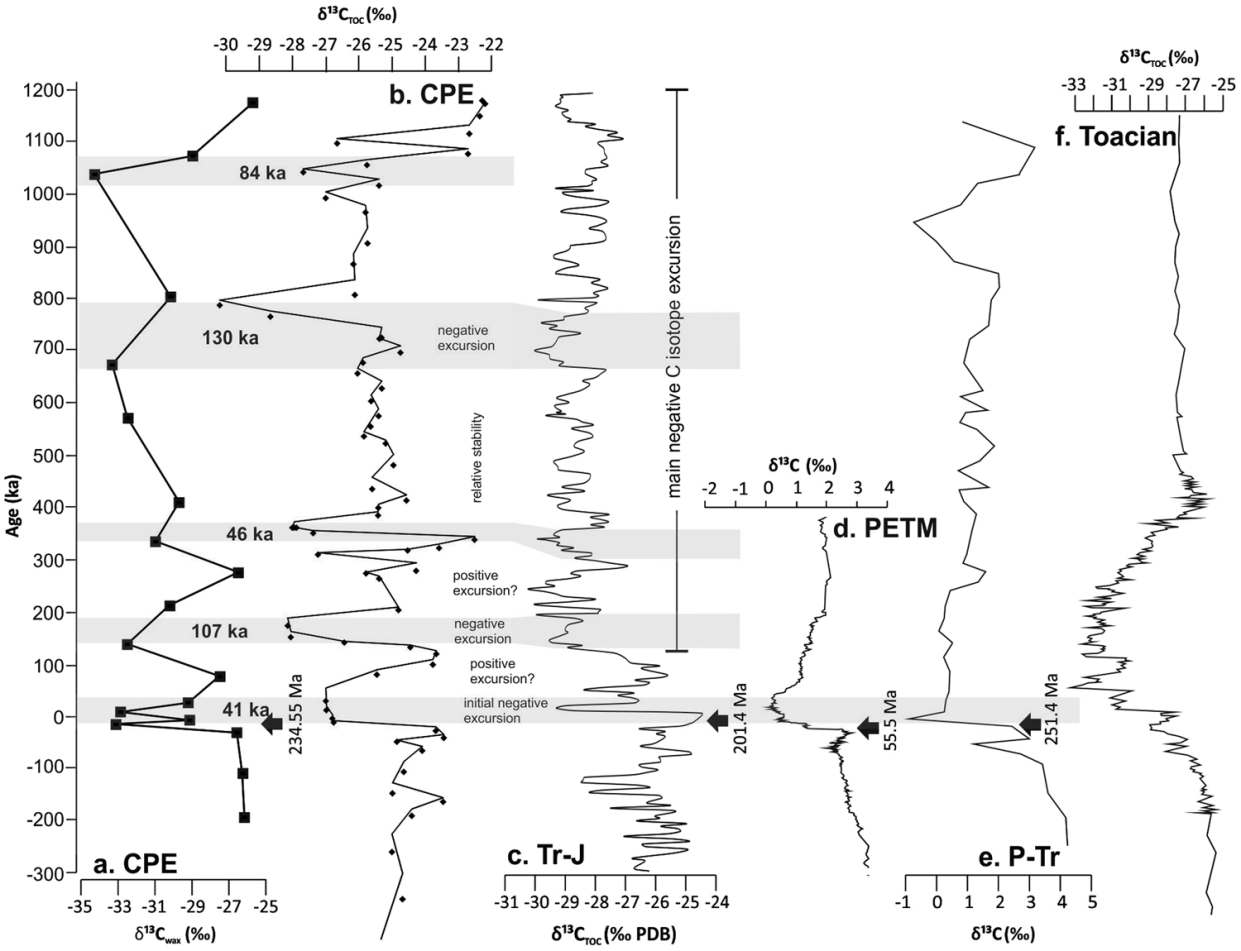
A comparison between the CPE negative CIE (this study) and other negative CIEs in the Phanerozoic. (**a**) Devon CPE δ^13^C_wax_ (this study). (**b**) Devon CPE δ^13^C_TOC_ (this study). (**c**) St Audrie’s Bay End Triassic Extinction (ETE) δ^13^C variations in organic matter^38, 39^. (**d**) δ^13^C record of marine bulk carbonate from ODP 690 over the Paleocene–Eocene Thermal Maximum (PETM)^42^. (**e**) End Permian δ^13^C record of marine bulk carbonate from southern China^40^. (**f**) Early Jurassic (Toarcian) δ^13^C variations in organic matter from Yorkshire (UK)^41^. 0 ka is set as the onset of all isotope excursions. The CPE onset is taken as the Julian 1–2 boundary at 234.55 Ma^4^. The floating chronology for the WP borehole 1 is formed from the tuning of the Ca/Ti data to the 405 ka orbital cycle (see Fig. 3).

Spectral analysis of the Ca/Ti x-ray fluorescence (XRF) elemental abundance and GR data^10^ reveals statistically significant regular cycles throughout the lacustrine DMF for both WP borehole 1 & 2 (Fig. 2d–e; Supplementary Fig. S6). Within the British Keuper series, rhythmically alternating patterns in sedimentation were also identified elsewhere e.g. ref. 21 and 22. Implementing the frequencies (MTM and F-test) from the Ca/Ti and the GR dataset into the average spectral misfit (ASM) gives a sedimentation rate of 0.02 mm year^−1^ (1.9 cm ka^−1^; Fig. 3a and b), indicating that the dominant cyclicity present at *c*. 8 m likely corresponds to the 405 ka eccentricity cycle (Fig. 3a and b). This sedimentation rate is comparable to similar arid environments within the Late Triassic such as at St Audrie’s Bay (UK) and in the Germanic Basin at *c*. 0.012–0.016 mm year^−1^ ^23, 24^. In the Newark Basin, the full range of orbital cycles is observed in Late Triassic lacustrine successions, where sedimentation rates of 0.16 mm year^−1^ are approximately an order of magnitude greater than at WP (Fig. 1)^25^. On the Yangtze Platform (South China Block) Carnian climate oscillations are related to long and short eccentricity cycles as well as precessional variability^26^. Cyclicity is less clear within the XRF data of the SMF (Fig. 2d–e) and in the GR data in the overlying Branscombe Mudstone Formation (BMF)^10^ at WP. Moreover, no conclusive evidence is found for the 405 ka eccentricity cycles within the BMF at St Audrie’s Bay^23^. Caliche concretions and frosted sands suggest that these homogeneous red mudstones of the SMF and BMF were likely deposited in a hyper-arid environment^12^, which is unfavourable for recording orbital cyclicity. Breaks in sedimentation and erosional surfaces likely cause the observed loss in cyclicity within these formations. Nevertheless, the recognition of 405 ka eccentricity cycles through the DMF in Devon allows us to establish a chronology (Fig. 3) and indicates that the whole negative C-isotope excursion (20.4 m; CPE) lasted for *c*. 1.09 Ma, with the IIE spanning just 41 ka (Figs 2–4). Importantly, our 1.09 Ma duration for the CPE is comparable to previous estimates of 0.8–1.2 Ma^26^.

Unlike the marine succession from the Dolomites where one CIE is identified^3^, we identify a further four appreciable negative CIE’s within the CPE (IIE *c*. δ^13^C_wax_ 6–7‰; Fig. 2). The ‘missing’ isotope excursions in the Dolomites^3^ and Austrian^4^ successions is perhaps the result of sampling resolution differences. Siliciclastic pulses are indeed evident in the Dolomites and in the Northern Calcareous Alps, and provide evidence for humid-arid environmental variability throughout the western Tethys realm during the CPE^7^. In view of the large amplitude of the isotope excursions several sources of isotopically light carbon must be considered to identify the underlying causes, including: a collapse in primary productivity, magmatically derived CO_2_, volatilized organic carbon from buried biomass and methane. Triassic *p*CO_2_ values are estimated at *c*. 4500 ppmv, and thus the atmosphere likely contained *c*. 9540 Gt C^27^. Source mixing analysis^28^ allows us to estimate the amount of C (*n*) required from each source to explain the observed negative CIE: (9540 Gt C + *n* Gt C) × (δ^13^C_atmosphere_ early Julian II) = 9540 Gt C × (δ^13^C_atmosphere_ late Julian I) + *n* Gt C × (δ^13^C_emission_). Our calculations indicate that even a complete collapse in primary productivity (modern biomass *c*. 830 Gt C, δ^13^C = *c*. −20‰^1^) would only account for around one quarter of the carbon required (*c*. 3500 Gt C) to produce the observed δ^13^C isotope shift. It is plausible that the CPE was associated with a crisis in marine bioproductivity, further supported by the existence of a widespread demise in reef ecosystems^2, 4, 6, 17^; however, insufficient evidence for such an extensive collapse exists. The CPE excursion would require an additional 14,000 Gt C of magmatically derived CO_2_ (using δ^13^C = *c*. −8‰^29^). However pools of lighter carbon in the mantle, as well as peridotic and eclogitic diamonds, have been measured with δ^13^C values as low as −38–−22‰^30, 31^. If the δ^13^C value is set to −20‰, then only *c*. 3500 Gt C is required to produce our observed isotope excursion. With estimates of 5000 Gt C released during the eruption of the Wrangellia igneous province alone^3^, a pure volcanic scenario is possible. Alternatively, if volatilized organic carbon was the primary source of the light-C, then *c*. 2700 Gt C would have been required to produce the observed excursion (δ^13^C = *c*. −25‰^32^), the equivalent of all present-day terrestrial carbon stocks^33^. Nevertheless, there is little evidence for substantial contact metamorphism of carbon-rich sediments within petroleum-bearing basins during the CPE. Conversely, just a small contribution (*c*. 1000 Gt C) from methane clathrates (δ^13^C = *c*. −60‰^34^) would have been required to produce the observed excursion. Estimates indicate that marine sediments at present contain about 10,000 Gt of methane carbon, thus a release of 1000 Gt C is plausible^35^. The stability of methane hydrates is temperature dependent, with warming leading to destabilisation. Indeed, we observe evidence for climatic warming during the CPE at sites from the eastern and northwestern Tethys^5, 17, 36^. Such methane release would quickly be sequestered by both terrestrial and marine components of the global carbon cycle, and would contribute to the widespread deposition of the black shales evidenced during the CPE^5^.

Volcanic emissions from the Wrangellia igneous province and the dissociation of methane clathrates could independently account for the observed multiple negative CIE’s within CPE. Nevertheless, a causal relationship likely exists between the Wrangellia eruption and the subsequent destabilization of methane clathrates. It seems that a combination of both volcanic emissions and the dissociation of methane clathrates would be the most likely explanation for the substantial observed shift(s) in δ^13^C, a scenario comparable to that of the End Triassic Extinction (ETE)^37^. We envisage episodic, punctuated release of methane from clathrates during progressive warming caused by the Wrangellia eruptions.

Multiple similarities exist, between the C isotope excursion observed at the CPE and ETE (Fig. 4a–c ^37–39^), for example: (i) a substantial IIE (6–7‰ CPE, 2–4‰ ETE), (ii) a similar duration of IIE (41 ka CPE, 20–40 ka ETE), (iii) a negative positive couplet (108 ka CPE, 80 ka ETE), and (iv) a return to oscillating but generally depleted values throughout the excursion. The end Permian excursion shows again a sharp, short initial excursion, however the swing back toward heavier values is much smaller than at the CPE and ETE, and values remain some 0–1.5‰ lighter (Fig. 4e)^40^. This difference is explained by low surface productivity in the aftermath of the extinction, which suggests that productivity did not substantially decrease after the IIE of the CPE and ETE. Both the PETM and Toarcian C isotope excursions appear more gradual in nature, with the entire Toarcian excursion comprising four separate negative shifts each of 2–3‰ (Fig. 4d and f)^41, 42^.

Compound specific and total organic carbon δ^13^C analysis of Late Triassic (Carnian) sediments from Devon (UK) provide the first evidence from non-marine strata of the negative CIE observed, coinciding with the CPE extinction event. Furthermore, in contrast to marine successions, we identify not one, but five, significant isotope excursions. By utilizing the persistent presence of strong 405 ka eccentricity cycles through the 20.4 m record of the DMF we have constructed an astronomical timescale for 1.09 Ma years of the Late Triassic, allowing us to estimate a likely duration for the C-isotope excursions associated with the CPE. Through source mixing analysis we calculate that a combination of volcanic emissions and subsequent methane release were the likely cause for the observed shift in δ^13^C and the associated extinction event.

## Methods summary

Sediments from WP borehole 1 were extracted with dichloromethane (DCM):MeOH (9:1, v/v) using soxhlet extraction. The total extract was separated into apolar and polar fractions over an alumina oxide column, eluting with hexane:DCM (9:1, v/v) and DCM:MeOH (1:1, v/v), respectively. Compound δ^13^C was analysed using an Agilent 6890 gas chromatographer coupled with a Thermo Finnigan Delta_PLUS_XL isotope ratio mass spectrometer (GC-C-IRMS). Ratios were calibrated daily based on the reference standard Schimmelmann B. For δ^13^C_TOC_ analyses, samples from WP borehole 1 were decalcified, neutralized and subsequently dried. Samples were measured for % TOC using a Fisons NA1500 NCS and coupled with a Thermo Delta plus IR-MS for stable C-isotope analyses. Ratios were normalised using the laboratory standard GQ to the V-PDB standard. Major element abundances were attained using a hand-held Niton XRF analyser at the BGS. MTM, F-test, bandpass and ASM were performed on the XRF data from WP borehole 1 and GR data from WP borehole 2 using the R program Astrochron^43^. The Continuous Wavelet Transform was achieved using a Morlet wavelet and run on a MATLAB platform. Further technical information for all methods is provided in the supplementary information.

## Electronic supplementary material

Supplementary information
