# Supplementary material for: Astronomical age constraints and extinction mechanisms of the Late Triassic Carnian crisis
Source: Sci Rep. 2017 May 31;7:2557. doi: 10.1038/s41598-017-02817-7 (PMC5451402; doi:10.1038/s41598-017-02817-7)
Supplement: Supplementary file 1 — Supplementary information [file 41598_2017_2817_MOESM1_ESM.pdf]

Supplementary material and methods for:

Astronomical age constraints and extinction mechanisms of the Late Triassic Carnian crisis

Charlotte S. Miller, Francien Peterse, Anne-Christine Da Silva, Viktória Baranyi, Gert J. Reichart, Wolfram M. Kürschner

Compound specific C isotopes

Rocks from Wiscombe Park (WP) borehole 1 were cleaned with methanol and ground with a pestle and mortar. Subsequently, the organic compounds were extracted from *c*. 15 g of the freeze-dried, homogenized rock sample with 200ml of dichloromethane (DCM):MeOH (9:1, v/v) using soxhlet extraction. The total lipid extracts were dried under N_2 and elemental sulphur was removed using copper turnings. The total extract was separated into apolar and polar fractions by passing them over an alumina oxide column, eluting with hexane:DCM (9:1, v/v) and DCM:MeOH (1:1, v/v), respectively. For $\delta^{13}C_{wax}$ the apolar fraction was dried under N_2 and hexane was added before analysis using the GC-MS and the GC-IR-MS at Utrecht University. The identification of higher plant-derived *n*-alkanes was achieved using the GC-MS reference library and through comparison to the reference *n*-alkane standard. Compound $\delta^{13}C$ was analysed in duplicate (σ = 0.04-4.5, ave. 0.8, see Table S1) using an Agilent 6890 gas chromatographer coupled with a Thermo Finnigan $Delta_{PLUS}XL$ isotope ratio mass spectrometer (GC-C-IRMS). Separation of compounds was achieved using a non-polar column CP-Sil 5 CB (25 m, 0.32mm internal diameter). The GC oven temperature was programmed from 70°C to 130°C at a rate of 20°C/min, then from 130°C to 280°C (rate: 4°C/min) and finally held at 280°C for 20 min. Ratios were calibrated daily based on the reference standard Schimmelmann B (purchased from A. Schimmelmann, Biogeochemical Laboratories, Indiana University). A characteristic chromatogram of the extracted apolar fraction is given in Fig. S1. Values are reported as the weighted mean of *n*-C_{27}–*n*-C_{35} alkanes.

The **average chain length** (ACL) was calculated for C_{27}–C_{33} *n*-alkanes using the following equation:
ACL = (27*A27+29*A29+31*A31+33*A33)/(A27+A29+A31+A33)

The **carbon preference index** (CPI) was calculated for C_{25}–C_{34} *n*-alkanes using the following equation:
CPI = (((A25+A27+A29+A31+A33)/(A26+A28+A30+A32+A34)) +
((A25+A27+A29+A31+A33)/(A26+A28+A30+A32+A34)))*0.5

*Where A in both equations is the area under the chromatographic peak for each *n*-alkane of a specific chain length in the GC chromatogram.

The extracted long chain *n*-alkanes from WP have CPIs ranging from 1.7–4.2, with average values of 2.5, similar to other Late Triassic CPI values calculated from the Eiberg Basin[1], the Dolomites[2], the Newark and Hartford Basin and St Audrie's Bay[3] (Table S2). Higher plant leaf waxes characteristically have odd-over-even *n*-alkane chain lengths (e.g. Fig. S1), leading to CPI values >5. Low CPI values indicate higher degradation. The freshwater algae *Botryococcus* has a similar *n*-alkane distribution to higher plants[4]. Nevertheless, preliminary and ongoing palynological analyses indicate that *Botryococcus* is not present within these samples, mitigating its contribution to the plant-derived *n*-alkane signal. Consequently, the long-chain *n*-alkanes found within the WP borehole are likely to derive from higher plant leaf-waxes. Isotope values of individual high molecular weight *n*-alkanes and average chain lengths (ACL) are shown in Fig. S2.

Numerous factors can influence the carbon isotopic composition of higher plant *n*-alkanes such as humidity and soil moisture availability, temperature, altitude, changes in plant type, pCO_2, $\delta^{13}C_{atm}$, light exposure and nutrient availability, with most studies based on modern plant material[5,6,7]. Moisture availability has been identified as the strongest single control on $\delta^{13}C_{wax}$ in modern systems[6] (assuming $\delta^{13}C_{atm}$ remains constant). The negative CIEs observed during the CPE correspond to a lithological change and a switch to hygrophytic flora, suggesting a widespread increase in precipitation[8,9]. In the modern system, $\Delta\delta^{13}C_{wax}$ (isotopic difference between $\delta^{13}C_{atm}$ and $\delta^{13}C_{wax}$) tends to increase with increasing water availability[7,10]. Nevertheless, a shift from dry conditions (<500mm yr^{-1}) to humid conditions (>1000mm yr^{-1}) would for example result in a $\Delta\delta^{13}C_{wax}$ of *c*. 4‰[7], which would not account for the here observed CIEs. It is therefore unlikely that a precipitation change alone is the driver for the $\delta^{13}C_{wax}$ variations during the CPE. Secondly, we observe evidence for climatic warming during the CPE[11]. With studies of C_3 plants showing positive correlations between $\delta^{13}C_{wax}$ and temperature[12], temperature is again unlikely to be the underlying cause of the excursion. Increased volcanism is evident during the CPE[2], which likely resulted in an increase in pCO_2 and changes $\delta^{13}C_{atm}$. If the atmosphere becomes saturated with CO_2 (e.g. during the CPE *c*. >4000 ppmv), then the $\Delta\delta^{13}C_{wax}$ associated with increasing pCO_2 is close to 0‰[7,13], making it unlikely that isotopic shifts during the CPE were related to changes in pCO_2. We therefore conclude that the CIEs recorded in both $\delta^{13}C_{wax}$ and $\delta^{13}C_{TOC}$ were most likely caused by the injection of volcanic light carbon into the atmosphere, which changed the $\delta^{13}C_{atm}$.

Source mixing analysis

For the source mixing analysis we assume that lipid $\delta^{13}C$ is *c*. 10‰ lighter than whole plant tissue $\delta^{13}C$[14]. However, variations in isotopic fractionation can be caused by changes in climate and plant type[5]. As our location is tropical, and greater fractionation is generally observed in tropical and subtropical plants, we use the value of 10‰ as a maximum[15].

We reconstruct an estimate of the $\delta^{13}C$ record of the atmosphere using the average $\delta^{13}C_{wax}$ and the empirically derived relationship ($\delta^{13}C_{atmosphere}=[\delta^{13}C_{plant}+18.67]/1.10$)[16] (Fig. S3). It should be noted however that recent studies have shown that environmental factors (temperature, water availability) as well as plant species shifts can impact the utility of $\delta^{13}C_{plant}$ to predict $\delta^{13}C_{atmosphere}$[5, 17, 18].

$\delta^{13}C$ of TOC and % TOC

For $\delta^{13}C_{TOC}$ analyses, 78 sample aliquots from WP borehole 1 were homogenized and treated sequentially with 0.1M and 1M HCl for 24 hours, before being rinsed to neutrality with MilliQ water (18.2 MΩ cm) and drying at 40°C for 4 days. Each step, involving a change of reagent or water, was preceded by centrifugation (10 min at 1500 rpm) to prevent the loss of fine material in suspension. The resulting powders were weighed into tin cups. Samples were first measured for % TOC using a Fisons NA1500 NCS and then for isotopes using the Fisons NA1500 NCS coupled with a Thermo Delta plus IR-MS (Table S3). Ratios were normalised using the laboratory standard GQ (a powdered Graphite-Quartzite). The precision obtained for repeat analysis was better than ± 0.15‰ (σ). A comparison between the equivalent samples measured for $\delta^{13}C_{TOC}$ and $\delta^{13}C_{wax}$ show good covariance and a positive correlation (Fig. S4).

Major elemental analysis

Five hundred and sixteen samples from WP borehole 1 were analysed for major element abundances using a hand-held Niton XRF analyser at the British Geological Survey (UK). For the elements key to this study (Ca & Ti) the calibration errors associated with these measurements were better than 0.02% for Ca and 0.07% for Ti.

Palynostratigraphy

The chronostratigraphy of the continental Mercia Mudstone Group and its sub-units, which includes the DMF and the SMF, is hampered by the scarcity of biostratigraphic indicator fossils[19]. Previous palynological studies indicate a Carnian age for the DMF and SMF[19,20,21,22,23]. However, our new palynological data allow us to further improve the age assessment for the studied interval (Fig. S5). The DMF contains typical Carnian palynoflora including *Camerosporites secatus*, *Duplicisporites granulatus*, *Aulisporites astigmosus*, *Triadispora verrucata*, *Partitisporites* spp. and *Paracirculina granifer*[9,24,25,26,27,28]. The upper part of WP borehole 1 (56.51m and above) is likely to be Julian 2 in age. It contains the first appearance datum of species such as *Patinasporites densus*, *Enzonalasporites vigens* and *Partitisporites maljawkinae, P. novimundanus and P. quadruplicus*. *Partitisporites* spp. is characteristic for the *densus-maljawkinae* phase[28], which correlates with the

Lagenella martini assemblage zone[26] and the *austriacus* zone indicating late Julian age (Julian 2). The lower part of the core (SMF) is exceptionally poor in pollen with most samples barren, likely due to the oxidation of organic matter in a hyper-arid depositional setting. Only two samples provided an exceptionally rich palynological assemblage. *Triadispora verrucata* was recorded from this interval which indicates early Julian age (Julian 1)[26, 27]. *Aulisporites astigmosus* occurs within the SMF and within one sample in the DMF (Fig. S5). The characteristic *A. astigmosus* acme[9,24,26,27] is completely missing from the WP borehole. However, as this acme typically occurs in fluvial deposits, we explain the lack of *A. astigmosus* as a likely environmental and taphonomical phenomenon. With the substantial IIE correlating well with the lithological change to a significantly wetter depositional environment (as seen globally), we associate the change from the SMF to DMF to the Julian 1–2 transition.

Cyclostratigraphy

Interpolation, detrending, MTM and F-test[29], bandpass and average spectral misfit (ASM)[30] were performed using the R program Astrochron[31]. Spectra were calculated on linearly detrended and interpolated data using multi-taper spectral analysis (2 tapers used, as outlined in[32]) in Astrochron. Confidence intervals were also determined in Astrochron using the robust methodology of [33], based on a second order polynomial fit to the median smoothed spectra. The Continuous Wavelet Transform[34] was performed using a Morlet wavelet and run on a MATLAB platform. The ASM compares the frequencies obtained through spectral analysis reaching 95% CL, with the orbital frequencies for the Triassic[35] and computes the sedimentation rate that provides the best fit, with the highest confidence level. MTM and F-test, statistically important peaks for the spectral analysis of the XRF datasets are shown in Fig. S6. ASM was used as an objective estimation of the optimal sedimentation rate for the stratigraphic interval spanning the DMF (Fig. 3a & b).

Through the DMF the Ca/Ti XRF elemental data shown in Fig. S6 reveals a cycle averaging around 800 cm with high statistical significance. Additionally, significant cycles are present in the Ca/Ti data at *c*. 75, 40 and at 25 cm. To perform ASM the minimum sedimentation rate was set at 0.005 cm ka^{-1} and maximum set at 3 cm ka^{-1}. These boundaries were obtained through a comparison of the succession thickness with an estimation of the duration of the interval (compared to the 2012 Geological Time Scale[36]), and through comparison with sedimentation rates in analogous Triassic depositional settings[37,38]. The number of sedimentation rates to investigate was set at 100. The optimal sedimentation rate was found to be 1.9 cm ka^{-1} for both Ca/Ti and gamma ray records, with a H_o-significance level of 0.18% (meaning that there is only a 0.18% probability that such an ASM value is derived by chance) for Ca/Ti and 3.91% for GRS. The raw Ca/Ti dataset was bandpassed, for the 405-ka cycle, based on the likely ASM generated sedimentation rate, with the lowest frequency set at 1/8.4 and the highest at 1/7 m^{-1} (shown in red on Fig. 2e). The Ca/Ti record was tuned through

multiple tie-points, related to the 405 ka cycle at a frequency of 0.13 m^{-1} (interpreted from the sedimentation rate of 1.9 cm ka^{-1}); which resulted in a record in the time domain instead of the distance domain (Fig. 3c–f). The wavelet transform of this tuned record show clearly the 405 ka cycle, but also show a clear cycle around ~100 ka, modulated by the 405 ka cycle (Fig. 3d & f).

Supplementary figures and tables

Figure S1. Characteristic chromatogram of an extracted apolar fraction (sample at 72.95 m). Note odd-over-even pattern in higher plant *n*-alkanes. Note the high abundance of mid-chain *n*-alkanes in this sample, possibly a result in increased sediment maturity, decreasing the *n*-alkane average chain length of the sample[39]. Only long chain (>C_{27}), un-decomposed alkanes were used for data interpretation.

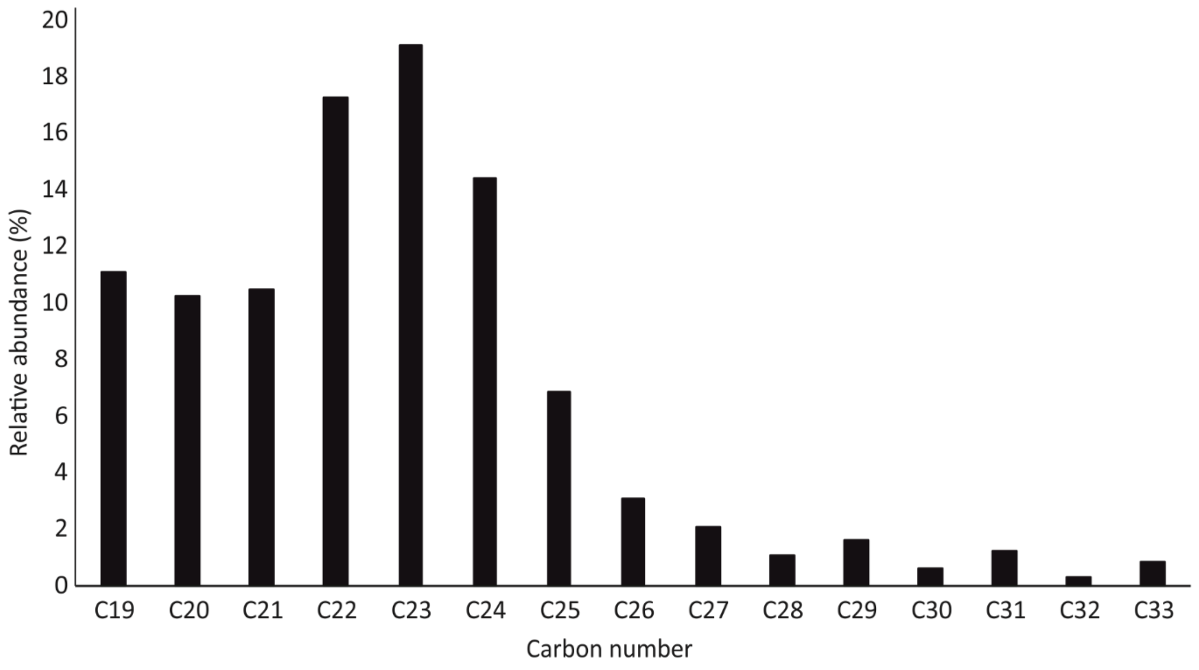

Figure S2. A comparison between the $\delta^{13}C$ of high molecular weight *n*-alkanes (C_{27}-C_{33}) and their average chain length (ACL) from WP borehole 1 sediments over the CPE.

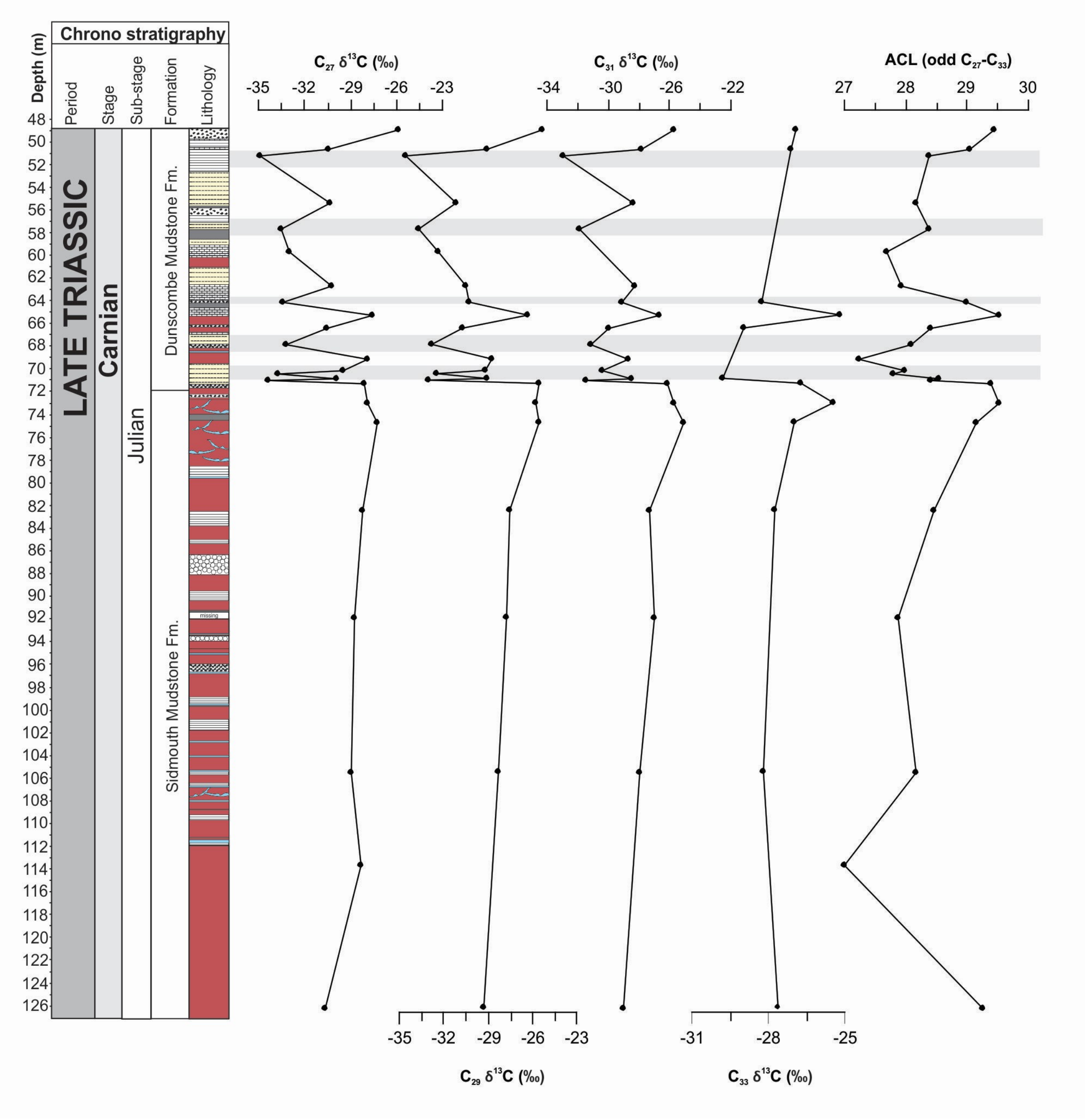

Figure S3. Calculated $\delta^{13}C$ of atmospheric CO_2 using $\delta^{13}C_{atmosphere}=[\delta^{13}C_{plant}+18.67]/1.10)$[16, 40].

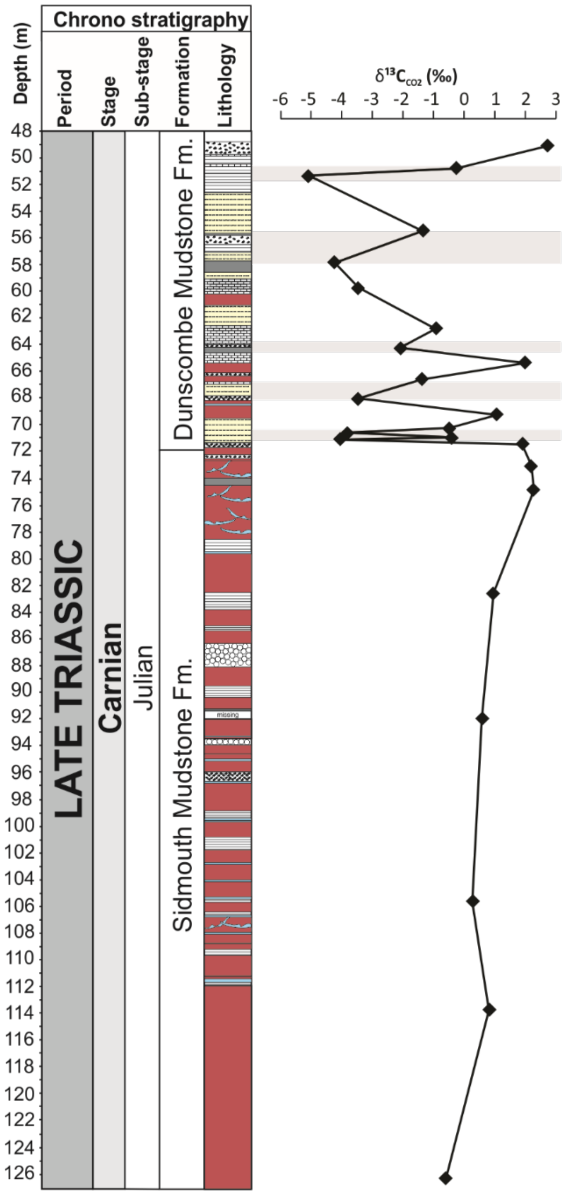

Figure S4. a) A comparison between the $\delta^{13}C_{TOC}$ and $\delta^{13}C_{wax}$ at WP showing only the $\delta^{13}C_{TOC}$ where equivalent samples were measured for $\delta^{13}C_{wax}$. Note the similar pattern of change through the CPE. The five negative isotope excursions are shown as blue bars. b) The relationship between $\delta^{13}C_{TOC}$ and $\delta^{13}C_{wax}$.

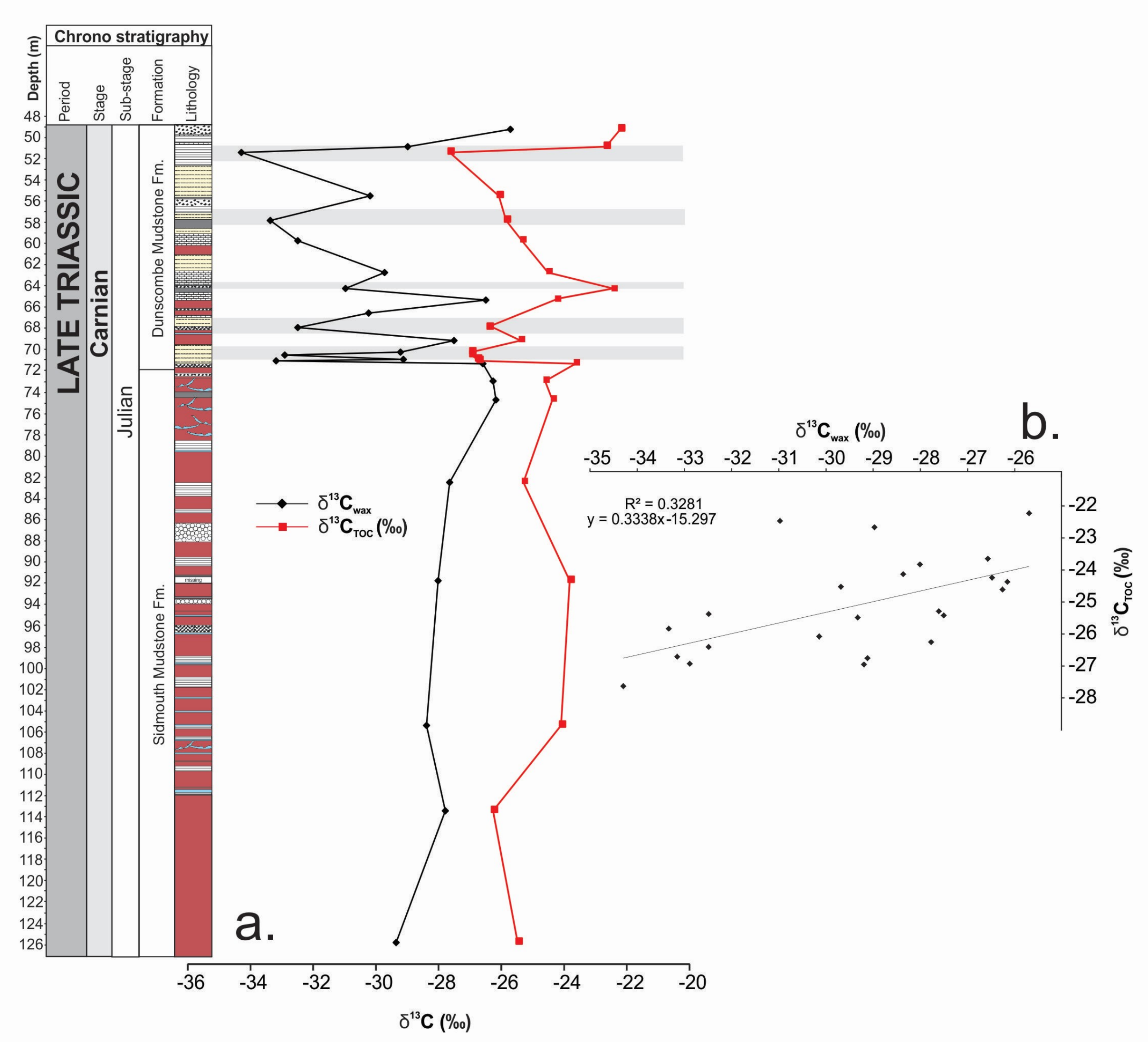

Fig. S5. Palynoflora occurrences in WP borehole 1. Note black lines are recorded occurrences and dashed are extended stratigraphic ranges.

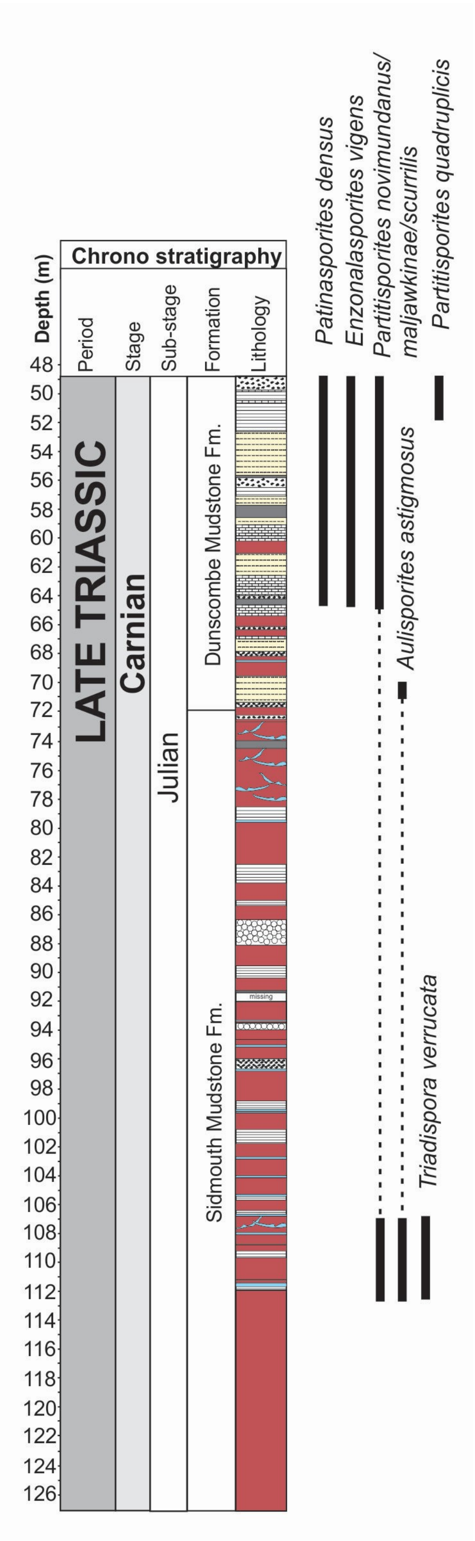

Figure S6. Spectral analysis of the Ca/Ti XRF elemental abundance data from WP borehole 1 and gamma ray data from WP borehole 2 revealing evidence for cyclicity throughout the DMF at *c*. 766 and 727 cm respectively. Additionally, significant cycles are present in the Ca/Ti data at *c*. 75 cm, 40 cm and at 25 cm. The filtered version of the Ca/Ti dataset (filter frequency = 0.13 m^{-1}, i.e. 8 m wavelength) is shown in Fig. 2, highlighting the statistically verified variability and supporting the inference that the *c*. 800 cm cycles likely correspond to 405 ka orbital cyclicity. Grey bars fitting with significant peaks are associated with an approximate age based on the calculated likely sedimentation rate from ASM (1.9 cm ka^{-1}).

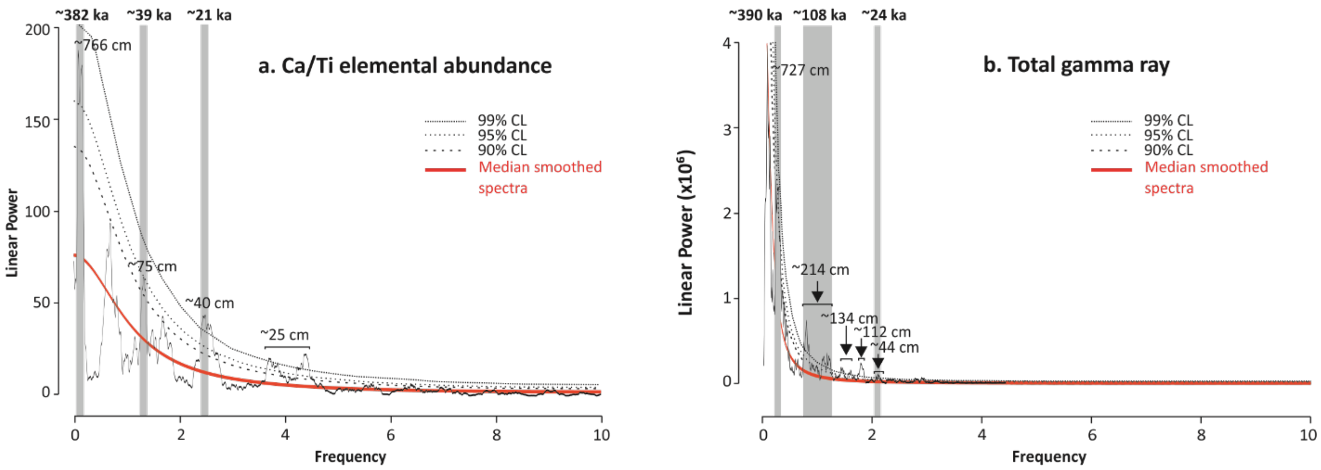

Table S1. C-isotope values for long-chain *n*-alkanes C_{27}-C_{35} from WP.

Depth (m)	CPI	$\delta^{13}C_{C27}$ (‰)	$\delta^{13}C_{C29}$ (‰)	$\delta^{13}C_{C31}$ (‰)	$\delta^{13}C_{C33}$ (‰)	$\delta^{13}C_{C35}$ (‰)
48.94	3.5	-25 ± 0.6	-25 ± 0.5	-26 ± 1.3	-27 ±1	-26 ± 0.4
50.58	2.0	-30 ± 0.4	-29 ± 1.8	-28 ± 0.5	-27 ± 0.8	-28[b]
51.17	3.2	-34 ± 0.6	-35 ± 0.0	-33 ± 0.2	-[a]	-[a]
55.30	2.1	-30 ± 0.6	-31 ± 1.0	-28 ± 0.3	-[a]	-[a]
57.68	2.0	-33 ± 0.6	-34 ± 1.0	-32 ± 0.3	-[a]	-[a]
59.59	3.0	-33 ± 0.9	-32 ± 0.2	-[a]	-[a]	-[a]
62.65	2.3	-30 ± 1	-30 ± 2.6	-28 ± 0.0	-[a]	-[a]
64.08	1.9	-33 ± 1	-30 ± 1.0	-29 ± 0.6	-28 ± 0.2	-[a]
65.23	2.7	-27 ± 0.2	-26 ± 1.1	-27 ± 0.5	-25 ± 1.2	-[a]
66.46	2.5	-30 ± 0.1	-31 ± 1.0	-30 ± 1.3	-29 ± 0.2	-[a]
67.91	4.2	-33 ± 0.2	-33 ± 0.3	-31 ± 0.7	-[a]	-[a]
69.13	2.4	-27 ± 0.4	-29 ± 1.6	-29 ± 0.9	-[a]	-[a]
70.14	2.0	-29 ± 0.6	-29 ± 1.1	-30 ± 1	-[a]	-[a]
70.50	2.7	-33 ± 4.2[c]	-32 ± 4.5[c]	- [a]	-[a]	-[a]
70.83	2.5	-29 ± 0.2	-29 ± 0.2	-28 ± 0.2	-30 ± 0.5	-[a]
70.98	2.9	-34 ± 0.1	-33 ± 0.6	-31 ± 0.3	-[a]	-[a]
71.31	2.3	-28 ± 0.4	-26 ± 1.0	-26 ± 0.4	-27 ± 0.8	-[a]
72.95	2.5	-27 ± 0.7	-26 ± 0.5	-26 ± 0.7	-26[b]	-[a]
74.76	2.1	-27 ± 0.5	-26 ± 0.4	-25 ± 0.4	-27[b]	-[a]
82.54	3.3	-28 ± 0.3	-28 ± 0.9	-27 ± 0.4	-28 ± 0.4	-[a]
91.94	1.7	-28 ± 0.0	-28 ± 0.4	-27 ± 0.6	-[a]	-[a]
105.68	1.9	-28 ± 0.3	-28 ± 1.7	-28 ± 0.8	-28 ± 0.9	-[a]
113.83	2.5	-28 ± 0.8	-[a]	-[a]	-[a]	-[a]
126.45	2.2	-30 ± 1.5	-29 ± 0.0	-29 ± 0.7	-28 ± 1.4	-[a]

-[a] Not determined due to low *n*-alkane concentration
[b] Measurement based on one sample
[c] Note high standard deviation

Table S2. A comparison between Late Triassic CPI values.

Study	CPI value range
Dolomites[2]	1.6–2.3
St Audrie's Bay[41]	0.41–2.55
Eigberg Basin[1]	1.3–4.2
Devon (this study)	1.7–4.2

Table S3. Total organic carbon (TOC) $\delta^{13}C$ data. Grey highlighted boxes are replicated samples.

**Depth (m)**	% TOC	$\delta^{13}C_{TOC}$
48.94	0.6	-22.2
49.03	0.3	-22.1
49.41	0.9	-22.3
49.95	1.6	-22.6
50.58	0.5	-22.7
50.25	0.4	-26.6
50.94	0.7	-25.7
51.17	0.7	-27.6
51.60	0.2	-25.3
52.01	0.6	-27.0
52.48	0.1	-25.8
53.52	0.1	-25.7
54.23	0.1	-26.1
55.30	0.1	-26.1
56.79	0.2	-25.3
56.83	0.2	-25.3
57.32	0.1	-24.7
57.68	0.1	-25.8
58.06	0.1	-26.0
58.60	0.1	-25.3
59.06	0.1	-25.6
59.59	0.1	-25.4
59.97	0.1	-25.6
60.33	0.1	-25.8
60.58	0.1	-25.1
61.37	0.1	-24.9
62.23	0.1	-25.6
62.65	0.1	-24.5
62.92	0.1	-25.4
63.20	0.1	-25.4
63.64	0.4	-27.9

63.64	0.4	-28.0
63.64	0.4	-27.8
63.84	0.4	-27.3
64.08	0.4	-22.5
64.39	0.3	-23.5
64.48	0.5	-24.5
65.23	0.1	-24.2
65.52	0.1	-25.3
65.32	0.2	-25.7
66.69	0.1	-24.8
67.27	0.2	-28.1
67.70	0.3	-28.0
67.91	0.3	-26.4
68.08	0.1	-24.4
68.34	0.1	-23.6
68.75	0.2	-23.7
69.13	0.1	-25.4
70.14	0.1	-27.0
70.50	0.1	-26.9
70.83	0.1	-26.8
70.98	0.2	-26.7
71.31	0.2	-23.6
71.59	0.2	-23.4
71.74	0.2	-24.8
72.10	0.1	-24.0
72.95	0.1	-24.6
73.83	0.1	-24.9
74.18	0.2	-23.4
74.76	0.1	-24.4
76.31	0.1	-25.0
78.22	0.1	-24.6
82.54	0.1	-25.3
89.57	0.2	-23.5
91.94	0.1	-23.8

98.90	0.1	-24.6
103.44	0.1	-25.3
103.32	0.1	-24.2
105.68	0.1	-24.1
109.11	0.3	-25.7
111.70	0.1	-25.8
111.98	0.2	-24.7
113.83	0.1	-26.3
114.59	0.1	-26.4
126.45	0.1	-25.5
126.45	0.1	-25.5
126.45	0.1	-25.8

References

1. Ruhl, M., Bonis, NR,. Reichart, G-J., Damsté, J.S.S., Kürschner, W.M. Atmospheric Carbon Injection Linked to End-Triassic Mass Extinction. *Science* **333**, 430-434 (2011).

2. Dal Corso, J. *et al.* Discovery of a major negative δ13C spike in the Carnian (Late Triassic) linked to the eruption of Wrangellia flood basalts. *Geology* **40**, 79-82. (2012).

3. Whiteside, J.H., Olsen, P.E., Eglinton, T., Brookfield, M.E., Sambrotto, R.N. Compound-specific carbon isotopes from Earth's largest flood basalt eruptions directly linked to the end-Triassic mass extinction. *Proceedings of the National Academy of Sciences* **107**, 6721-6725 (2010).

4. Lichtfouse, É., Derenne, S., Mariotti, A., Largeau, C. Possible algal origin of long chain odd n-alkanes in immature sediments as revealed by distributions and carbon isotope ratios. *Organic Geochemistry* **22,** 1023-1027 (1994).

5. Diefendorf, A.F., Freimuth, E.J. Extracting the most from terrestrial plant-derived n-alkyl lipids and their carbon isotopes from the sedimentary record: A review. *Organic Geochemistry* **103,** 1-21 (2017).

6. Diefendorf, A.F., Mueller, K.E., Wing, S.L., Koch, P.L., Freeman, K.H. Global patterns in leaf 13C discrimination and implications for studies of past and future climate. *Proceedings of the National Academy of Sciences* **107,** 5738-5743 (2010).

7. Schubert, B.A., Jahren, A.H. The effect of atmospheric CO2 concentration on carbon isotope fractionation in C3 land plants. *Geochimica et Cosmochimica Acta* **96,** 29-43 (2012).

8. Gallois, R.W. The stratigraphy of the Mercia Mudstone Group succession (mid to late Triassic) proved in the Wiscombe Park boreholes, Devon. *Geoscience in south-west England : proceedings of the Ussher Society* **11**, 280-286 (2008).

9. Fisher, M.J. The Triassic Palynofloral Succession in England. *Proceedings of the Annual Meeting American Association of Stratigraphic Palynologists* **3**, 101-109 (1972).

10. Farquhar, G.D., Ehleringer, J.R., Hubick, K.T. Carbon Isotope Discrimination and Photosynthesis. *Annual Review of Plant Physiology and Plant Molecular Biology* **40**, 503-537 (1989).

11. Sun, Y.D. *et al.* Climate warming, euxinia and carbon isotope perturbations during the Carnian (Triassic) Crisis in South China. *Earth and Planetary Science Letters* **444,** 88-100. (2016).

12. Wang, G., Li, J., Liu, X., Li, X. Variations in carbon isotope ratios of plants across a temperature gradient along the 400 mm isoline of mean annual precipitation in north China and their relevance to paleovegetation reconstruction. *Quaternary Science Reviews* **63,** 83-90 (2013).

13. Treydte, K.S. *et al.* Impact of climate and CO2 on a millennium-long tree-ring carbon isotope record. *Geochimica et Cosmochimica Acta* **73,** 4635-4647 (2009).

14. Marshall, J.D., Brooks, J.R., Lajtha, K. Sources of Variation in the Stable Isotopic Composition of Plants. *Stable Isotopes in Ecology and Environmental Science* 22-60 (Blackwell Publishing Ltd 2008).

15. Freeman, K.H., Pancost, R.D. Biomarkers for Terrestrial Plants and Climate in *Treatise on Geochemistry (*ed Turekian, K.K.) 395-416 (Elsevier: Oxford, 2014).

16. Arens, N.C., Jahren, A.H., Amundson, R. Can C3 Plants Faithfully Record the Carbon Isotopic Composition of Atmospheric Carbon Dioxide? *Paleobiology* **26**, 137-164 (2000).

17. Jahren, A.H., Arens, N.C. Prediction of atmospheric δ13CO2 using plant cuticle isolated from fluvial sediment: Tests across a gradient in salt content. *PALAIOS* **24,** 394-401 (2009).

18. Lomax, B.H., Knight, C.A., Lake, J.A. An experimental evaluation of the use of C3 δ13C plant tissue as a proxy for the paleoatmospheric δ13CO2 signature of air. *Geochem Geophys Geosyst* **13** (2012).

19. Howard, A., Warrington, G., Ambrose, K., Rees J.A. formational framework for the Mercia Mudstone Group (Triassic) of England and Wales. *British Geological Survey Research Report* (2008).

20. Warrington, G. The stratigraphy and palaeontology of the 'Keuper' Series of the central Midlands of England. *Quarterly Journal of the Geological Society* **126**, 183-223 (1970).

21. Warrington, G. The Lyme Regis Borehole, Dorset - palynology of the Mercia Mudstone, Penarth and Lias groups (Upper Triassic - Lower Jurassic). *Proceedings of the Ussher Society* **9**, 153-157 (1997).

22. Gallois, R.W., Porter, R.J. The stratigraphy and sedimentology of the Dunscombe Mudstone Formation (late Triassic) of south-west England. *Geoscience in south-west England* **11,** 174-182 (2006).

23. Porter, R.J., Gallois, R.W. Identifying fluvio–lacustrine intervals in thick playa-lake successions: An integrated sedimentology and ichnology of arenaceous members in the mid–late Triassic Mercia Mudstone Group of south-west England, UK. *Palaeogeography, Palaeoclimatology, Palaeoecology* **270**, 381-398 (2008).

24. Dunay, R.E., Fisher, M.J. The Karnian palynoflora succession in the Northern Calcareous Alps, Lunz am See, Austria. *Pollen et Spore* **20**, 177-187 (1978).

25. Kürschner, W.M., Herngreen, G.F.W. Triassic palynology of central and northwestern Europe: a review of palynofloral diversity patterns and biostratigraphic subdivisions. *Geological Society, London, Special Publications* **334**, 263-283 (2010).

26. Roghi, G., Gianolla, P., Minarelli, L., Pilati, C., Preto, N. Palynological correlation of Carnian humid pulses throughout western Tethys. *Palaeogeography, Palaeoclimatology, Palaeoecology* **290**, 89-106 (2010).

27. Fijałkowska-Mader, A., Heunisch, C., Szulc, J. Palynostratigraphy and palynofacies of the Upper Silesian Keuper (southern Poland). *Annales Societas Geologorum Poloniae* **85**, 637-661 (2015).

28. Van der Eem, J.G.L.A. Aspects of middle and late Triassic palynology. Palynological investigations in the Ladinian and lower Karnian of the Western Dolomites, Italy. *Review of Palaeobotany and Palynology* **39**, 189-300 (1983).

29. Thomson, D.J., Spectrum estimation and harmonic analysis. *Proceedings of the IEEE* **70**, 1055-1096 (1982).

30. Meyers, S.R., Sageman, B.B. Quantification of deep-time orbital forcing by average spectral misfit. *American Journal of Science* **307**, 773-792 (2007).

31. Meyers S.R. Astrochron: An R Package for Astrochronology (2014).

32. Weedon, G.P. Time Series Analysis and Cyclostratigraphy: Examining Stratigraphic Records of Environmental Cycles (Cambridge University Press 2003).

33. Mann, M., Lees, J. Robust estimation of background noise and signal detection in climatic time series. *Climatic Change* **33**, 409-445 (1996).

34. Torrence, C., Compo, GP. A Practical Guide to Wavelet Analysis. *Bulletin of the American Meterological Society* **79**, 61-78 (1998).

35. Berger, A. Astronomical theory of paleoclimates and the last glacial-interglacial cycle. *Quaternary Science Reviews* **11**, 571-581 (1992).

36. Ogg, J.G. Chapter 25 – Triassic in *The Geologic Time Scale*. 681-730 (Elsevier Boston, 2012).

37. Kemp, D.B., Coe, A.L., A nonmarine record of eccentricity forcing through the Upper Triassic of southwest England and its correlation with the Newark Basin astronomically calibrated geomagnetic polarity time scale from North America. *Geology* **35**, 991-994 (2007).

38. Vollmer, T. *et al.* Orbital control on Upper Triassic Playa cycles of the Steinmergel-Keuper (Norian): A new concept for ancient playa cycles. *Palaeogeography, Palaeoclimatology, Palaeoecology* **267**, 1-16 (2008).

39. Bray, E.E., Evans, E.D. Distribution of n-paraffins as a clue to recognition of source beds. *Geochimica et Cosmochimica Acta* **22**, 2-15 (1961).

40. Berner, R.A. Atmospheric Carbon Dioxide Levels Over Phanerozoic Time. *Science* **249**, 1382-1386 (1990).

41. Whiteside, J.H., Olsen, P.E., Kent, D.V., Fowell, S.J., Et-Touhami, M. Synchrony between the Central Atlantic magmatic province and the Triassic–Jurassic mass-extinction event? *Palaeogeography, Palaeoclimatology, Palaeoecology* **244**, 345-367 (2007).
